# The Strength of Alliance in Individual Psychotherapy and Patient's Wellbeing: The Relationships of the Therapeutic Alliance to Psychological Wellbeing, Satisfaction With Life, and Flourishing in Adult Patients Attending Individual Psychotherapy

**DOI:** 10.3389/fpsyt.2022.827321

**Published:** 2022-01-31

**Authors:** Tomasz Prusiński

**Affiliations:** Department of Personality Psychology, Institute of Psychology, The Maria Grzegorzewska University, Warsaw, Poland

**Keywords:** therapeutic alliance, psychological well-being, life satisfaction, flourishing, pantheoretical model of alliance, psychotherapeutic process, adult psychotherapy, structural equation models

## Abstract

**Objectives:**

The central aim of the research was to verify and determine the strength of the relationships of therapeutic alliance to wellbeing, life satisfaction, and flourishing in patients attending individual psychotherapy. The relationships were assessed based on different sources of information about the quality of the working alliance: patient's evaluation and patient's and psychotherapist's joint evaluations.

**Design:**

The author applied Bordin's pantheoretical model of alliance and two different conceptions of wellbeing, operationalized as hedonistic and eudaimonic.

**Methods:**

The 411 participants included 252 patients and 159 psychotherapists. To test the hypotheses, 16 joint and separate models of structural relations were built and analyzed empirically using SEM. Correlations were analyzed between alliance factors and those of wellbeing, satisfaction, and flourishing.

**Results:**

The actual impact of working alliance quality on psychological wellbeing proved to be stronger compared to the relations between alliance and satisfaction or flourishing. The results of analyses revealed low, though usually positive and significant, correlations between the dimensions of alliance and those of wellbeing, life satisfaction, and flourishing.

**Conclusions:**

The empirical data and the strategy of analyses brought the expected results, confirming that patient's and psychotherapist's perception of a strong therapeutic alliance is crucial for the optimization of patient's functioning and wellbeing. It turns out that the therapeutic alliance is, above all, a factor of wellbeing understood more deeply than merely as current pleasure. The study also showed that no factor isolated from other components of alliance increased the quality of patient's mental functioning more than others.

## Introduction

Previous research has established a belief that psychotherapy is an effective way of treating mental disorders and optimizing individuals'functioning (1–3). Less is known, however, about how psychotherapy works and what mechanisms are responsible for its outcomes (4, 5). Identifying the components of the therapeutic process that play the key role in recovery is important because it results in a better optimization of treatment and in a better understanding of the causal mechanisms that lead to disorders (6, 7). Researchers continue to investigate the significance of specific elements of psychotherapy and to identify its active components (8–10)—those that allow for achieving positive outcomes, operationalized both with objective indicators (the abatement of symptoms) and with subjective ones (an increase in patient's wellbeing) (11).

Studies on psychotherapy effectiveness are usually focused on reducing symptoms and interpersonal, cognitive, and social deficiencies in functioning (12). The improvement of overall quality of life is also often an implicit or explicit goal of psychotherapy. Frisch (13) defines the increase in quality of life or satisfaction with life as the subjective evaluation of the degree to which individual needs, goals, and wishes have been fulfilled. In psychotherapy, increased positive satisfaction with life or an improvement in wellbeing may be something more than merely a by-product of the alleviation of problems and symptoms: they may be an integral part of the transition from dysfunction to adaptation. Reporting a decrease in symptoms impairing a person's functioning is valuable, but it seems to be insufficient. The development of a healthy individual proceeds not only due to the decrease or absence of negative experiences or sensations, but also because the individual begins to experience themselves as a person having specific resources. It therefore seems important to determine what positive experiences, attitudes, and beliefs—generally, what change in wellbeing—an individual gains thanks to the process of psychotherapy.

### The Pantheoretical Model of Therapeutic Alliance

In the literature devoted to the issues of alliance in psychotherapy there is no single agreed-upon definition of the construct (14). The theory of the alliance commonly regarded as canonical is the one proposed by Bordin (15). Bordin calls the patient–therapist relationship the working alliance. The value of his model stems both from the essence of how alliance is understood in it and from the fact that it was thoroughly analyzed by the author of the construct and has been used in a number of research studies. In other words, the model has a strong empirical basis. This way of understanding the therapeutic relationship that has been adopted for the purposes of the current publication.

The working alliance can be defined as committed cooperation between patient and psychotherapist which is based on mutual trust and whose basic perspective is determined by the goals that have been agreed on and set to be pursued. It is emphasized that the working alliance is the most rational part of the patient–therapist relationship. A necessary condition for an alliance to emerge is that the patient must have a directed desire for recovery, some sense of helplessness or inadequacy, and a conscious need for cooperation with the psychotherapist.

Bordin suggests that alliance comprises three integrated components: agreement on goals, the assignment of tasks, and the development of bonds. The first two dimensions are specified at the initial meetings, which, for psychotherapists, are also sessions aimed at assessing the patient. The third dimension—though built during the entire period of meetings, as it is impossible to agree on mutual trust during the first sessions—is a condition of achieving the goals and performing the tasks. Bordin emphasizes that the quality of these three dimensions of the therapeutic alliance is what the success of psychotherapy and its short-term as well as final outcomes depend on. Thus understood, the alliance ensures the conditions necessary for the patient to build trust with respect to the proposed treatment, to accept it, and to adhere to the working rules agreed on in the further stages of psychotherapy.

### Therapeutic Alliance and Psychotherapy Outcomes

The main meta-analyses identifying the components of psychotherapy responsible for its positive outcomes (16) revealed no significant paths of relations. This finding induced some researchers to suspect that psychotherapy outcomes were unrelated to specific techniques used in various psychotherapeutic orientations and that they were more probably linked with non-specific, more universal factors common to different modalities of psychotherapy (17, 18). The classic results concerning the determinants of psychotherapy effectiveness, reported by Wilson and Lipsey (19), confirmed that the variance in the final outcome of psychotherapy was a product of non-specific factors.

A frequently considered factor of this kind is the therapeutic relationship (14, 20, 21). Also referred to in the literature as the therapeutic alliance (22, 23), “goodness of fit” between the therapist and the patient (24), the therapeutic partnership (25), or the working alliance (14, 15, 23), it is currently considered the most important determinant of effective psychological intervention, independent of psychologist's or psychotherapist's theoretical orientation (26). The therapeutic alliance is an important and powerful predictor of treatment outcomes, explaining an estimated 7.5% of the total variance in the outcomes of psychotherapy (20, 27).

The working alliance is considered to be an important determinant of psychotherapy success (28, 29) because it builds a framework for various methods and strategies used by psychotherapists. It builds communication between the psychotherapist and the stable part of patient's personality, helping the latter to remain in the process of change despite the fluctuating level of subjective discomfort or perceived difficulties in functioning. Establishing an optimal relationship with the patient enables the psychotherapist to adjust to those of the patient's characteristics that, for various reasons, could make it difficult to take a positive attitude toward them (30).

Studies indicate that a well-established therapeutic alliance is a determinant of positive treatment outcomes, on the condition that alliance is evaluated not only by the psychotherapist but also by the patient and that these evaluations coincide (21). Botella et al. (31) found that, with an increase in the number of sessions, the relationship between alliance and symptoms changed in such a way that a stronger alliance was accompanied by a decrease in symptoms. Likewise, the analyses performed by Zuroff and Blatt (32) indicatedthat the decrease in depressive symptoms was fasterin the patients who evaluated the quality of the therapeutic relationship as high.

Although the overall quality of life, wellbeing, and present life satisfaction are often both implicit and explicit aims of psychotherapy, there are few studies assessing such positive changes during treatment (12). Scarce empirical material is still cited that links the process of psychotherapy with outcomes in the form of life satisfaction and social or psychological wellbeing.

Frisch et al. (33) found a moderate relationship (0.42–0.57) of quality of life and wellbeing to treatment using psychotherapy. Seligman et al. (34) established that positive psychotherapy, aimed at increasing overall life satisfaction, led to greater changes in happiness among students with depression than ordinary treatment. There are also studies that go beyond the mental health context and suggest that the doctor–patient alliance, characterized by agreement on treatment goals and tasks and by mutual trust and liking, predict the maintenance of patients' present life satisfaction and an increase in their quality of life (35, 36).

Studies are lacking that would show that the isolated therapeutic alliance factor enhances quality of life—both temporarily (wellbeing) and in a more long-term perspective (further healthy development, psychological wellbeing, and flourishing). Looking for relations between the working alliance and wellbeing is consistent with the current paradigm of positive psychology, according to which wellbeing results from the dialectics of various positive and negative experiences or landmark moments in life (37). Although the psychotherapeutic alliance is the most often estimated determinant of success in psychotherapeutic treatment, little is known about the explanatory value of its components (38, 39).

The use of the concept of therapeutic alliance as a factor regulating the effects of psychotherapy with regard to the enhancement of wellbeing makes sense because it is supported by relational mechanisms (40) and by self-determination theory (41). The presence of alliance is not associated with demonstrating to patients that following recommendations or accomplishing tasks is good for their health and that it is in their best interest. A strong alliance, in turn, results in the development of a kind of non-instrumental social bond, based on respect for and trust in the proposed treatment and allowing for the acceptance of the recovery process (the bond effect). Finally, the way the alliance is implemented allows the individual to feel important and included in the decision-making process concerning their health (the agency effect). It should be expected that a properly built alliance, which is, by its very nature, based on a close relationship and on the autonomy of the patient's actions, will be reflected in the quality of his or her functioning. Based on the mechanisms outlined above, I therefore assumed that the alliance should enhance a positive and realistic attitude toward oneself, support the rebuilding of the patients' undermined agency, develop the ability of building deep relationships, and promote independence and self-directedness, thus reducing helplessness.

### The Present Study

The main aims of the study were:

1. to investigate the links and determine the strength of the relations of the therapeutic alliance to wellbeing, life satisfaction, and flourishing (12, 14, 36);
2. to check if these relations change depending on who is the source of information about alliance quality: the patient alone or both patient and psychotherapist (42, 43).

To accomplish these aims, I tested the following hypotheses:

H1: A higher quality of alliance reported by a patient leads to higher psychological wellbeing.

H2: A higher quality of alliance reported by a therapist–patient dyad leads to higher psychological wellbeing.

H3: A higher quality of alliance reported by a patient leads to higher satisfaction with life.

H4: A higher quality of alliance reported by a therapist–patient dyad leads to higher satisfaction with life.

H5: A higher quality of alliance reported by a patient leads to higher quality of functioning (flourishing) in life.

H6: A higher quality of alliance reported by a therapist–patient dyad leads to higher quality of functioning (flourishing) in life.

I tested which structural factor of alliance was the leading one in terms of impact on life satisfaction, wellbeing, and flourishing. The hypothesis was:

H7: The tasks assigned, the goals agreed on, and the psychotherapeutic bonds developed are positively correlated with the dimensions of wellbeing, life satisfaction, and flourishing.

In the present study, I relied on Bordin's pantheoretical model of alliance and on diverse approaches to wellbeing. Wellbeing was operationalized in two ways, derived from different philosophical traditions: hedonistic and eudaimonic. According to the former, wellbeing can be the experience of pleasure, contentment, and subjective satisfaction with life (44), while according to the latter it is the long-term experience that accompanies the fulfillment of one's potential and life in harmony with nature (45). Eudaimonic wellbeing is defined as the stable experience of optimal functioning manifesting itself in a positive attitude toward oneself, the ability to build deep relationships, a sense of autonomy, the ability to control one's environment, and having a firm belief about the direction of one's life.

**Figure 1:**
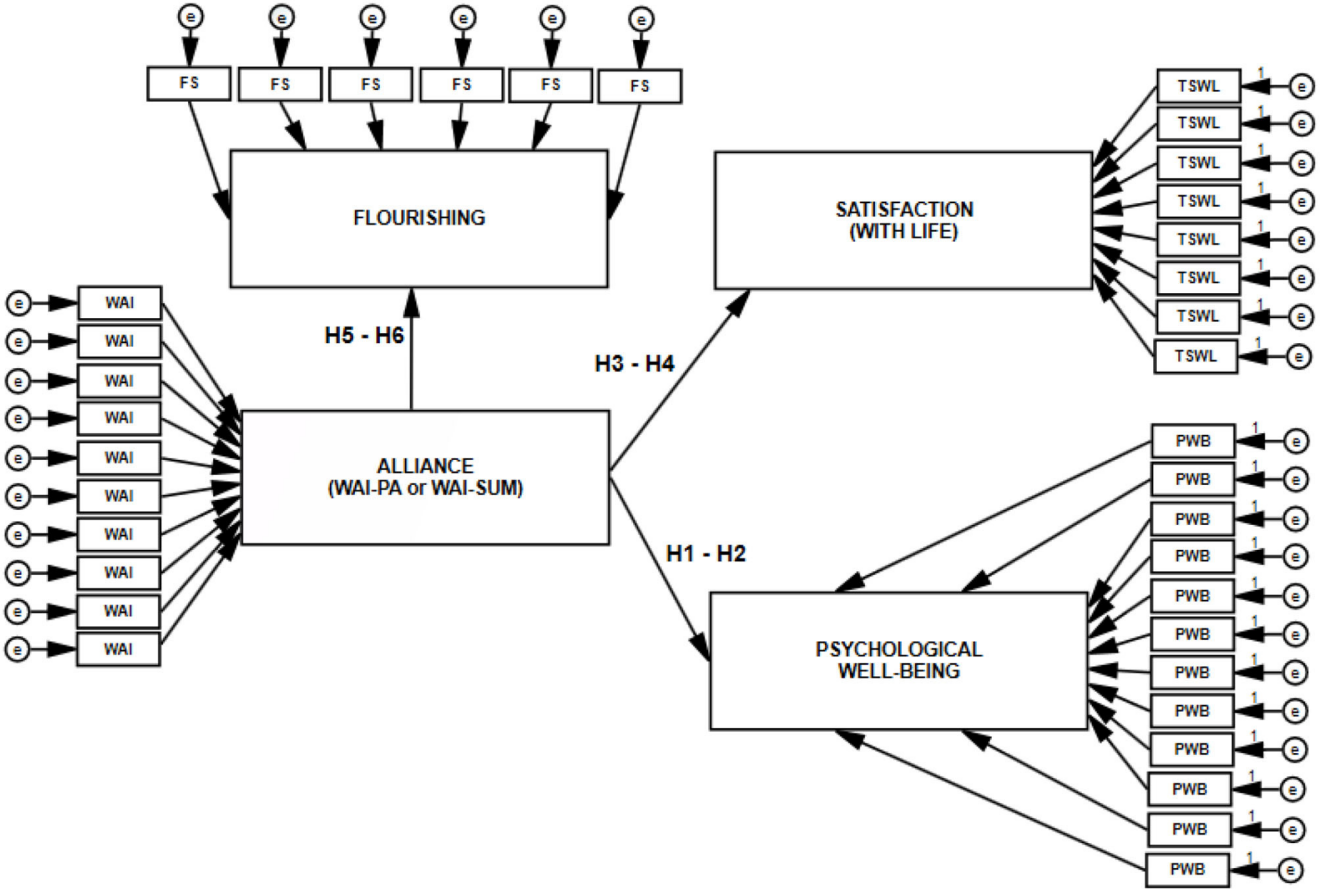
Model of direct relations of working alliance to well-being, life satisfaction, and flourishing. WAI-PA, isolated assessment of alliance based on patient's evaluation; WAISUM, assessment of alliance based on patient's and psychotherapist's evaluations; WAI, FS, TSWL, PWB, measures of the variables; e, random component.

**Figure 2:**
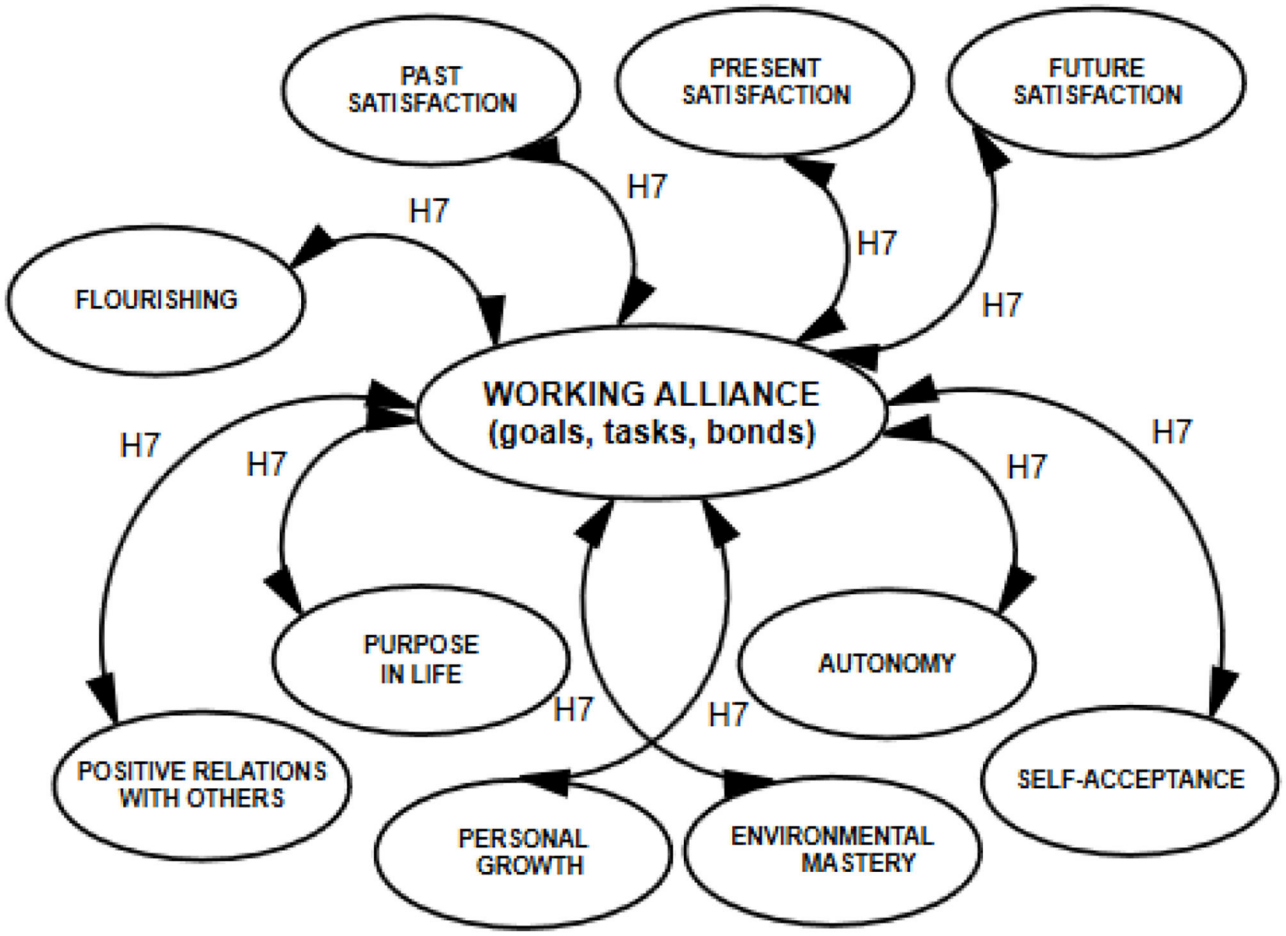
Model of correlations detailed for the dimensions of explanatory variables.

The model of hypothesized relationships among the analyzed constructs, verified based on empirical data, is shown in Figures 1, 2.

## Materials and Methods

### Participants and Procedure

Empirical research was conducted between February 2019 and June 2020. A total of 440 individuals were invited to take part in the study: 270 patients and 170 psychotherapists. The final sample consisted of 411 participants: 252 patients and 159 psychotherapists. In the study testing the relations between the variables, the assessment of therapeutic alliance and wellbeing was based on evaluations collected from 252 psychotherapist–patient dyads. The study dropout rate was 0.069 (6.9%) in the case of therapists and 0.071 (7.1%) in the case of patients.

Participation in the study was voluntary and anonymous. The participants—both patients and psychotherapist—were recruited from private and public psychotherapy offices. Information about the study was first given to the psychotherapist and then to the patient. The participants—the psychotherapist and the patient—were informed about the purpose of the study and asked to give their consent to take part in it. After granting the consent, the psychotherapist completed the Working Alliance Inventory and a survey sheet with questions about demographic variables and psychotherapeutic work context variables. The patient began with completing the Working Alliance Inventory and went on to complete a battery of scales concerning the evaluation of wellbeing and a sociodemographic survey. In this study, I analyzed data collected in a single measurement. The respondents received no remuneration for participation in the study.

### Statistical Methods

I used the SPSS 25 and IBM SPSS AMOS 25 statistical packages. Preliminary analyses of the participants' sociodemographic data and correlation analyses were performed by means of SPSS 25. To analyze SEM models, I used the AMOS 25 package.

### Measures

#### The Working Alliance Inventory

To assess working alliance quality, I used the full version of the WAI. The WAI is available in three versions: patient's version (WAI-PA), psychotherapist's version (WAI-PT), and a version estimating the working alliance by summing patient's and psychotherapist's evaluations (WAI-SUM) (46). Each version consists of 36 analogous items operationalizing the construct of working alliance, which the respondent rates on a Likert scale as accurately or inaccurately describing the cooperation in the patient–psychotherapist dyad being evaluated. The WAI score can be computed for three subscales; it is also possible to determine alliance quality by computing the total score. Each subscale is composed of 12 items: 6 positive and 6 negative ones.

In my study I administered two versions of the measure: WAI-PA and WAI-SUM. The WAI-PA was used because it was the patient's mental state and wellbeing that were estimated, which made it reasonable to ask about the patient's evaluation of the working alliance. The WAI-SUM was used because patient's and psychotherapist's weighted evaluation of alliance corrects the possible overestimations or underestimations that may occur when evaluation is performed exclusively by the patient.

The reliability of the total score is α_WAI−PA_ = 0.97 and α_WAI−SUM_ = 0.98, and for the subscales it is as follows: α_WAI−PA_ = 0.93 and α_WAI−SUM_ = 0.95 for Goals; α_WAI−PA_ = 0.93 and α_WAI−SUM_ = 0.95 for Tasks; α_WAI−PA_ = 0.93 and α_WAI−SUM_ = 0.96 for Bonds. CFA showed that measurement using the WAI was valid.

#### The Temporary Satisfaction With Life Questionnaire

The TSWLS (47) measures integrated evaluation of life as a whole that existed, continues to exist, and will exist. It consists of 15 items. The overall score is the sum of item scores. Respondents give their answers on a 7-point scale from *completely disagree* to *completely agree*. TSWLS scores were used to assess the short-term outcomes of psychotherapy, understood as the current sense of satisfaction or contentment.

#### The Psychological WellBeing Scale

This scale (48) consists of 18 items and measures long-term integrated psychological wellbeing as a whole. Respondents give their answers on a 6-point scale from *strongly disagree* to *strongly agree*. The measure allows for the estimation of six components of wellbeing: autonomy, environmental mastery, personal growth, positive relations with others, purpose in life, and self-acceptance. TSWLS and PWBS have good and very good reliability and validity (49).

#### The Flourishing Scale

The Flourishing Scale (FS) (50) is an 8-item measure of the quality of functioning in important domains, such as relationships, self-esteem, purpose, and optimism. It yields a single score (α_FS_ = 0.91). Respondents give their answers on a 7-point scale from *strongly disagree* to *strongly agree*.

The data that support the findings of this study are available on request from the corresponding author. The data are not publicly available due to privacy or ethical restrictions. The collection of data that served as the basis for the analyses performed was financially supported by state institutions and the university.

## Results

### Participants' Sociodemographic Characteristics

On the side of patients there were 252 Polish participants, including 129 women (51.2%) and 123 men (48.8%). Female participants were 17 to 80 years old (*M* = 35.37, *SD* = 11.81), and male participants' age ranged from 18 to 70 (*M* = 37.34, *SD* = 9.85). Most patients had higher (55.2%) or secondary education (42%) and lived in cities with a population above 100,000 (61.1%). In the whole sample, 86 participants (34.1%) were single and 166 (65.9%) were married or had a partner. By the time of the measurement, the patients had attended between 2 (1.2%) and 960 (0.4%) sessions (*M* = 37.01, *SD* = 82.71). A hundred and eighty-nine participants (75%) attended psychotherapy once a week, and most of the psychotherapeutic sessions (76.6%) took 50–60 min.

The type of disorder experienced by the participants in the group of patients was a variable controlled for to a limited degree. A few patients had more than one diagnosis; others were unable to give an unambiguous one. As regards the disorders that patients' were treated for, the largest group were individuals diagnosed with affective and mood disorders (32.9%). Mental and behavioral disorders caused by the use of alcoholic and psychoactive substances were diagnosed in 23.4% of patients and adaptation disorders were diagnosed in 13.5% of cases; 9.5% of psychotherapies were conducted due to personality disorders, schizophrenia, schizotypal disorders, and delusional disorders. In 7.5% of patients the reported reason for psychotherapeutic work was anxiety disorders and phobias, and 1.2% of patients needed psychotherapy due to an experience of trauma in the pretherapeutic period.

On the psychotherapists' side of the dyads, the participants were Polish, 109 women and 50 men, aged 27–64 (*M* = 42.98, *SD* = 9.48). The psychotherapists taking part in the study worked in the following modalities: psychoanalytic or psychodynamic (25.4%), cognitive-behavioral (31.7%), Ericksonian (12.3%), systemic (10.3%), humanistic (4.4%), and Gestalt (9.1%); 35.7% of therapies were conducted by psychotherapists with 1–5 years of work experience, while 63.9% were conducted by psychotherapists with more than 5 years of experience. 92.1% of the psychotherapists were doing or had completed at least 2-year training in psychotherapy; 52.7% had a certificate from a Polish psychotherapeutic associations.

### Preliminary SEM Analyses

**Figure 3:**
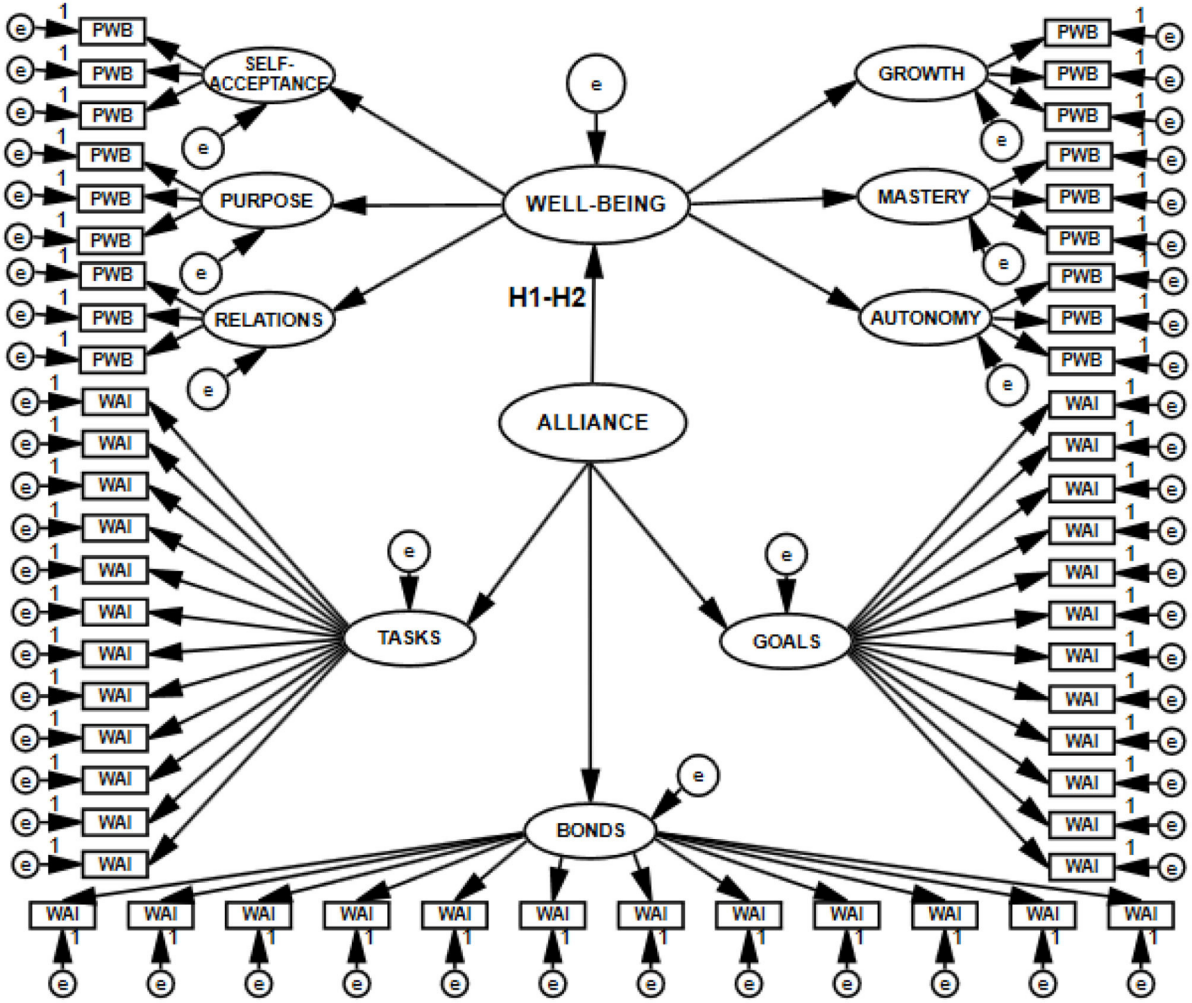
Structural and measurement model with 11 latent variables postulating the direction of relations between the working alliance and psychological well-being, tested with SEM.

**Figure 4:**
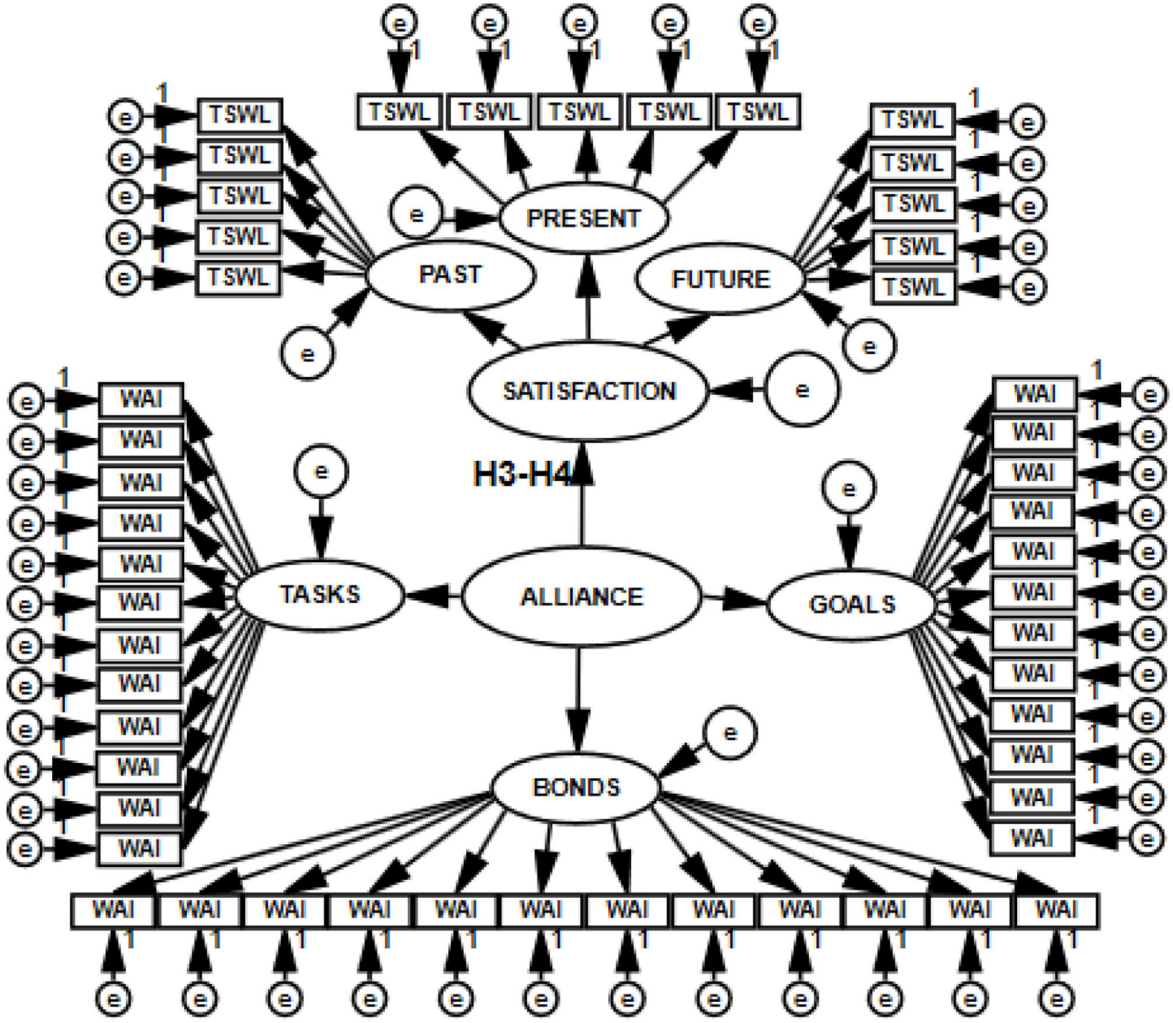
Structural and measurement model with eight latent variables postulating the direction of relations between the working alliance and satisfaction with life, tested with SEM.

**Figure 5:**
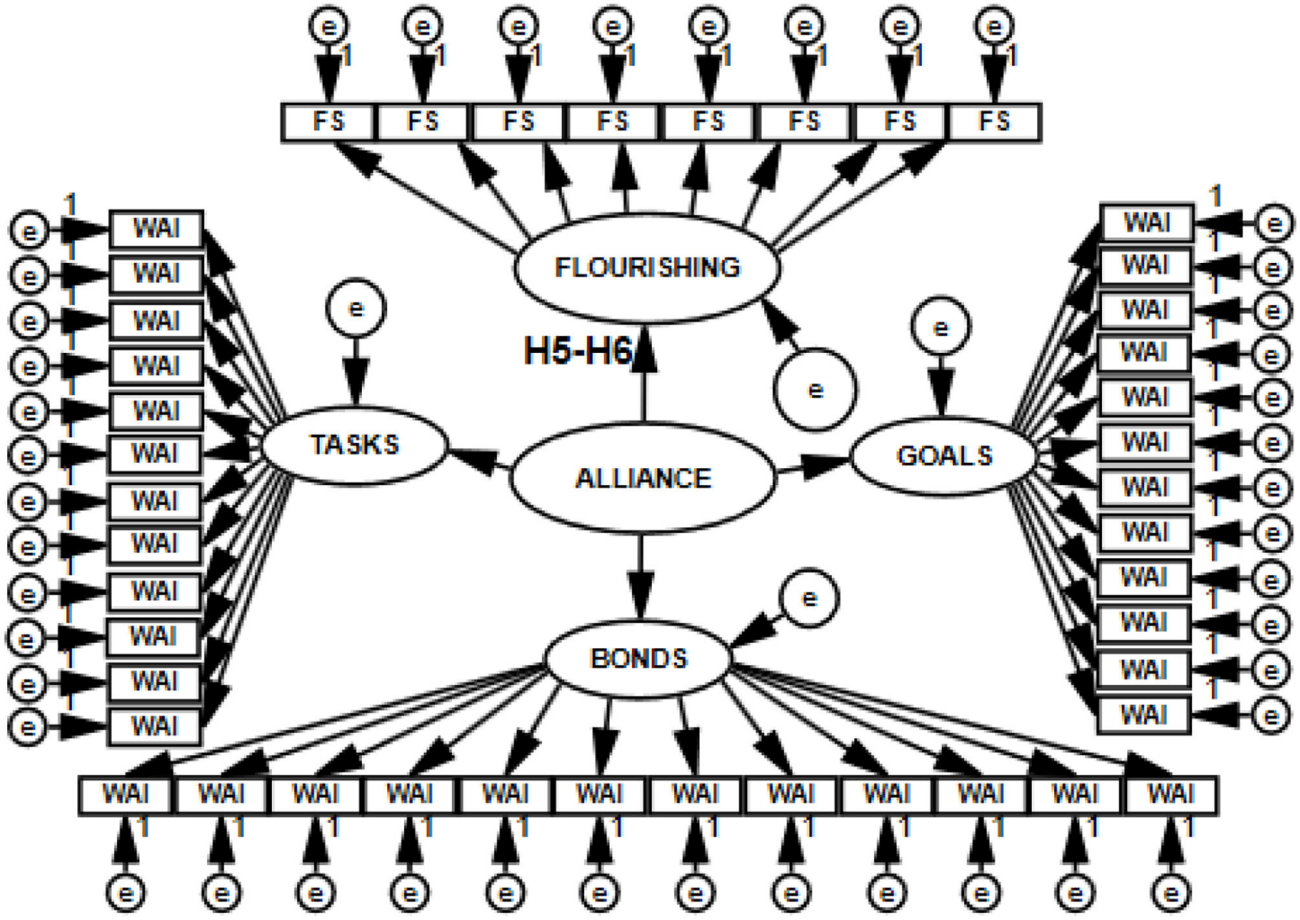
Structural and measurement model with five latent variables postulating the direction of relations between the working alliance and flourishing, tested with SEM.

**Figure 6:**
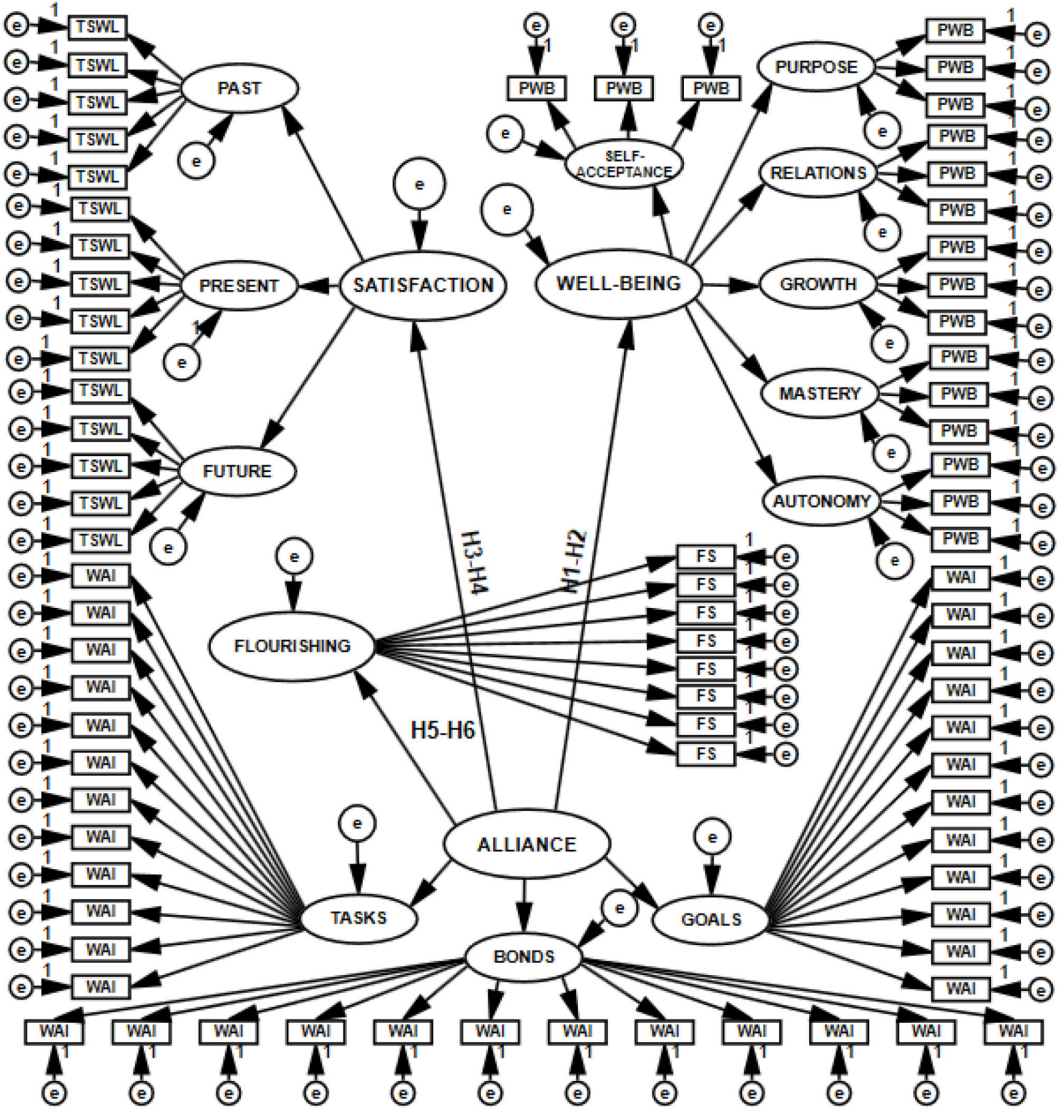
Structural and measurement model with 16 latent variables postulating the direction of the relations of the working alliance to psychological well-being, life satisfaction, and flourishing, tested with SEM.

To test the hypotheses (H1–H6), I built structural models with 11 latent variables (H1–H2), with 8 latent variables (H3–H4), with 5 latent variables (H5–H6), and with 16 latent variables (joint model for H1, H2, H3, H4, H5, and H6). Thus, I constructed four SEM models, which are presented in Figures 3–6.

To check if the relationships between the therapeutic alliance and the explained variables would change depending on who was the source of information about allianc equality, I changed the measurement model within the framework of the same structural model defining the relations for the latent variables entered. The measurement of working alliance was based either on the sum of patient's and psychotherapist's evaluations (WAI-SUM) or exclusively on the patient's evaluation (WAI-PA). Each of the SEM models was analyzed in the form of both full and simplified structures. A simplified model is unaffected by small sample size bias, which decreases the likelihood of the first type of error: rejecting a correct model. Such a model does not lose the postulated multidimensionality of the construct in any way (51). In the preliminary analyses estimating model fit, I tested 16 models. The results are presented in Table 1.

**Table 1:** Fit indices of the tested models.

**Hypotheses**	**SEM model**		**χ^2^**	***Df***	**χ^2^/*df***	***p***	**RMSEA**	**GFI**	**CFI**	**TLI**	**ECVI**	**MECVI**
H1, H2	WAI-PA	Full	3161.76	1367	2.31	0.01	0.072	0.65	0.81	0.80	13.54	13.80
	WAI-PA	Simplified	4.064	4	1.02	0.40	0.008	0.99	0.99	0.99	0.10	0.11
	WAI-SUM	Full	3357.36	1367	2.46	0.01	0.076	0.59	0.83	0.82	14.32	14.58
	WAI-SUM	Simplified	6.505	4	1.63	0.16	0.050	0.99	0.99	0.99	0.11	0.12
H3, H4	WAI-PA	Full	2432.14	1217	2.00	0.01	0.063	0.70	0.88	0.87	10.56	10.78
	WAI-PA	Simplified	22.22	8	2.78	0.01	0.084	0.97	0.91	0.83	0.19	0.19
	WAI-SUM	Full	2653.71	1217	2.18	0.01	0.069	0.65	0.88	0.88	11.44	11.67
	WAI-SUM	Simplified	12.04	8	1.88	0.06	0.059	0.98	0.96	0.92	0.16	0.17
H5, H6	WAI-PA	Full	1979.25	898	2.20	0.01	0.069	0.71	0.88	0.87	8.62	8.78
	WAI-PA	Simplified	2.14	4	0.53	0.71	0.001	0.99	0.99	0.99	0.10	0.10
	WAI-SUM	Full	2142.72	898	2.39	0.01	0.074	0.66	0.89	0.89	9.27	9.43
	WAI-SUM	Simplified	1.425	4	0.36	0.84	0.001	0.99	0.99	0.99	0.09	0.09
H1–H6	WAI-PA	Full	5,902.35	2834	2.09	0.01	0.066	0.59	0.77	0.76	24.86	24.47
	WAI-PA	Simplified	410.73	167	2.46	0.01	0.076	0.84	0.41	0.33	1.98	2.01
	WAI-SUM	Full	6091.26	2834	2.15	0.01	0.068	0.56	0.79	0.79	25.61	26.22
	WAI-SUM	Simplified	402.35	167	2.41	0.01	0.075	0.84	0.42	0.34	1.95	1.98

*χ^2^, chi^2^model fit statistic; df, degrees of freedom; χ^2^/df, chi^2^statistics divided by degrees of freedom; RMSEA= root mean square error of approximation; GFI, index of variance explained by the path model; CFI, comparative fit index; TLI, Tucker–Lewis index; ECVI and MECVI, information criteria for comparing the quality of models*.

Using the criteria for assessing fit indices (52, 53) for SEM models (χ^2^*/df* < 2.5; RMSEA ≤ 0.80; GFI and CFI values close to or exceeding 0.90; TLI values close to 0.95; ECVI and MECVI: the best model is considered to be the one for which the values are the lowest) and analyzing the values of the indices showing the fit of the theoretical model with the measurement model, I concluded that the models with very good and sometimes even excellent fit were simplified separate ones, estimating the relationship between alliance and each of the explained variables separately. Full separate models have acceptable or barely acceptable values of some of the fit indices (RMSEA, CFI, TLI). The joint structural models, including all relationships, demonstrate the poorest fit, though RMSEA values in their case are acceptable.

I therefore decided to consider all models when testing the hypotheses. The structural models are not alternative for one another, in the sense that they are not mutually exclusive. I expected that they would mutually support the relationships tested. Thus, the further verification of the hypotheses, based on results from multiple models, would rest on strong empirical support.

### Main SEM Analyses

The hypotheses postulating cause-and-effect relations between the working alliance as the explanatory variable and psychological wellbeing, life satisfaction, and flourishing as explained variables (H1–H6) were tested using structural equation modeling. SEM results are presented in Table 2.

**Table 2:** Standard estimators of the tested models.

**Models**	**Hypotheses**	**SEM model**			**β**	***p***	***R* ^2^**	***M* β**	***MR* ^2^**
Separate models	H1, H2	WAI-PA	Full		0.50	0.01	0.25	0.38	0.15
		WAI-PA	Simplified		0.34	0.01	0.12		
		WAI-SUM	Full		0.42	0.01	0.18		
		WAI-SUM	Simplified		0.26	0.01	0.07		
	H3, H4	WAI-PA	Full		0.28	0.01	0.08	0.29	0.08
		WAI-PA	Simplified		0.30	0.01	0.09		
		WAI-SUM	Full		0.28	0.01	0.08		
		WAI-SUM	Simplified		0.28	0.02	0.08		
	H5, H6	WAI-PA	Full		0.19	0.01	0.04	0.16	0.03
		WAI-PA	Simplified		0.20	0.01	0.04		
		WAI-SUM	Full		0.13	0.05	0.02		
		WAI-SUM	Simplified		0.13	0.03	0.02		
Joint (Comprehensive) models	H1–H6	WAI-PA	Full	WAI → Wellbeing	0.51	0.01	0.26		
				WAI → Satisfaction	0.30	0.01	0.09		
				WAI → Flourishing	0.21	0.01	0.04		
		WAI-PA	Simplified	WAI → Wellbeing	0.86	0.01	0.74		
				WAI → Satisfaction	0.70	0.01	0.49		
				WAI → Flourishing	0.66	0.01	0.43		
		WAI-SUM	Full	WAI → Wellbeing	0.42	0.01	0.18		
				WAI → Satisfaction	0.29	0.01	0.08		
				WAI → Flourishing	0.14	0.04	0.02		
		WAI-SUM	Simplified	WAI → Wellbeing	0.80	0.01	0.64		
				WAI → Satisfaction	0.66	0.01	0.44		
				WAI → Flourishing	0.58	0.01	0.33		

*β, standardized path coefficient; R^2^, multiple correlation coefficient; M β and MR^2^, mean values*.

The factor loadings were significant in each of the analyzed models. The actual effect of the working alliance quality on wellbeing proved to be the strongest (*Mβ*_SEPARATE SIMPLIFIED AND FULL MODELS_ = 0.38 and *Mβ*_JOINT SIMPLIFIED AND FULL MODELS_ = 0.65) compared to the effects between alliance and satisfaction (*Mβ*_SEPARATE SIMPLIFIED AND FULL MODELS_ = 0.29 and Mβ_JOINT SIMPLIFIED AND FULL MODELS_ = 0.49) or flourishing (*Mβ*_SEPARATE SIMPLIFIED AND FULL MODELS_ = 0.16 and *Mβ*_JOINT SIMPLIFIED AND FULL MODELS_ = 0.40). Likewise, the values of the multiple correlation coefficient *R*^2^ were the highest for the models that presented wellbeing as determined by alliance quality. The alliance–wellbeing models explain an average of 15% ($MR_{\text{SEPARATE MODELS}}^{2}$ = 0.15) to 45% ($MR_{\text{JOINT MODELS}}^{2}$ = 0.45) of the variance in the explained variable. These values are higher than the corresponding values for alliance–satisfaction ($MR_{\text{SEPARATE MODELS}}^{2}$ = 0.08, $MR_{\text{JOINT MODELS}}^{2}$ = 0.27) and alliance–flourishing models ($MR_{\text{SEPARATE MODELS}}^{2}$ = 0.03, $MR_{\text{JOINT MODELS}}^{2}$ = 0.20).

The results also show that the strength the relations between the therapeutic alliance and the explained variables slightly changes depending on who is the source of information about alliance quality. If the alliance is evaluated by the patient, the relationships are stronger (*Mβ*_SEPARATE PA MODELS_ = 0.30, *Mβ*_JOINT PA MODELS_ = 0.54) than when evaluations come from both sides of the alliance (*Mβ*_SEPARATE SUM MODELS_ = 0.25, *Mβ*_JOINT SUM MODELS_ = 0.48). In all of the analyzed structural conditions, these relations are positive.

To sum up, the values of coefficients yielded by SEM supported hypotheses H1, H2, H3, H4, H5, and H6. The six hypotheses were therefore accepted.

### Correlations

To test hypothesis H7, postulating positive relations between the dimensions of alliance (the assignment of tasks, agreement on goals, the development of bonds) and those wellbeing, life satisfaction, and flourishing, I performed correlation analyses. Table 3 presents the obtained results.

**Table 3:** Spearman's rho correlation coefficients between the dimensions of alliance and the dimensions of wellbeing, life satisfaction, and flourishing.

**Variable**		**Assignment of tasks**	**Agreement on goals**	**Development of bonds**
Satisfaction	Past	0.07	−0.10	−0.02
	Present	0.25^*^	0.20^*^	0.21^*^
	Future	0.22^*^	0.20^*^	0.22^*^
Psychological wellbeing	Self-acceptance	0.13^*^	0.10	0.11
	Purpose	0.05	0.06	−0.01
	Relations	0.28^*^	0.26^*^	0.21^*^
	Growth	0.21^*^	0.20^*^	0.16^*^
	Mastery	0.33^*^	0.34^*^	0.30^*^
	Autonomy	0.30^*^	0.26^*^	0.24^*^
Flourishing	Flourishing	0.21^*^	0.19^*^	0.18^*^

*PAST, satisfaction with the past life; PRESENT, satisfaction with the present life; FUTURE, satisfaction with the future life; PURPOSE, purpose in life; RELATIONS, positive relations with others; GROWTH, personal growth; MASTERY, environmental mastery*.

* *p < 0.01 (one-tailed)*.

The results of the analyses revealed low but mostly positive and significant correlations between the variables. This makes it reasonable to accept hypothesis H7.

All three working alliance factors correlate with flourishing and with present and future life satisfaction, but they do not correlate with the evaluations of satisfaction experienced in the past. The therapeutic alliance is associated with nearly all dimensions of psychological wellbeing. None of the dimensions of alliance is the leading one in terms of the number and strength of significant relations to the explained variables (*M*__*rho*_TASKS_ = 0.24, *M*__*rho*_GOALS_ = 0.24, *M*__*rho*_BONDS_ = 0.23).

## Discussion

The analyses presented in this study explored the relationships of the non-specific and universal factor in psychotherapy (17, 18), the alliance, to the important though not always explicit aims of psychotherapy: wellbeing, life satisfaction, and flourishing. In the current study I focused on the fact that, during psychotherapy, an individual may experience themselves as a person having developmental potential, and on the fact that the active factor in psychotherapy—the therapeutic alliance—may be related to this potential.

The collected empirical data and the strategy of testing the research objectives brought the expected results, confirming that patient's and psychotherapist's perceptions of a strong therapeutic alliance is crucial for the optimization of patient's functioning and wellbeing.

Importantly, the research plan in which alliance measurement based on patient's separate evaluation was enhanced with weighted estimation elicited from the recruited patient–psychotherapist dyad provides a strong empirical basis for conclusions. Supplementing separate evaluation with joint evaluation—elicited from two individuals: patient and psychotherapist—is also theoretically justified. An important characteristic of Bordin's working alliance is the mutuality of agreement. The strength of the alliance in this model is built by mutual consent to the actions undertaken and by maintaining a relationship of cooperation. The indicators of change are the goals achieved through specific tasks, which is possible thanks to the bond created between patient and psychotherapist. Therefore, if the alliance stems from the active participation of both individuals involved in the therapeutic process, then it is reasonable to take the opinion of both parties into account in its evaluation.

Introducing psychotherapist's evaluation of allianceis valuable, considering the potential limitations of the present study, such as the fact that the psychiatric symptoms experienced by the patients or the type of pharmacotherapy may have influenced their evaluation of the alliance and wellbeing. Supplementing the analyzed models with psychotherapist's evaluation of alliance ensured a correction of the patient's underestimations or overestimations in this regard. One should still be careful, however, when using the results of the analyses and conclusions presented in this study.

Alliance is a correlate of the maintenance of patients' wellbeing, present satisfaction, and flourishing. Of all the dimensions considered, I have identified those that alliance is most strongly related to. An improvement in psychological wellbeing accompanied by a strong alliance proved to be the main finding. It turns out that the therapeutic alliance is, above all, a factor of wellbeing understood more deeply than merely as current pleasure.

The present study supports the conclusions reached before by teams who analyzed the relations between the alliance and positive outcomes of psychotherapy (12, 21), confirming that these relations are increasingly positive with an increase in correspondence between patient's and psychotherapist's evaluations of the alliance. It also extends the previous findings by indicating that the relationships of working alliance to wellbeing, present satisfaction, and flourishing remain positive and significant if the alliance is evaluated by the patient alone. Compared to earlier analyses (33), the present study revealed similar and sometimes (as in the case of wellbeing) higher values indicating the strength of these relationships.

The study also showed that no factor isolated from other components of alliance increased the quality of patient's mental functioning more than others (38, 39) and that at the level of the analyzed components of alliance these relations were rather weak.

Importantly, however, the results of analyses showed significant associations between the dimensions of the therapeutic alliance (agreement on goals, the assignment of tasks, and the development of bonds) and the dimensions of wellbeing. High working alliance quality is accompanied by an increase in current life satisfaction. Also the future is perceived by the patient as more pleasant and its conditions as more acceptable. A strong therapeutic relationship is not related to the patient putting their past in order so as to make sure that it is no longer a source of suffering and negative feelings in the present life. This is an important finding, which allows for concluding that the process of psychotherapy improves present functioning and, possibly, makes it possible to discover resources and develop strategies to optimize future functioning. The “strong alliance effect” is not a factor motivating for changing the past. This may stem from the fact that the past is treated by the patient as a temporally closed space impossible to change. The association found in the study can also be explained as showing that the working alliance is not a consultative or advisory relationship, which means it does not consist in the psychotherapist indicating actions to be performed (54), and that patients themselves may be focused on improving their current health condition rather than on putting the past in order in an appropriate and satisfying manner. This result is consistent with the popular assumption about what psychotherapy is. Since, as Haley (55) pointed out, the main aim of psychotherapy is for people to start functioning appropriately to the reality in which they currently live, efforts associated with revising their emotional attitude to the past do not necessarily have to be a condition of successful psychotherapy.

As regards the associations of alliance dimensions with the dimensions of psychological wellbeing, it should be noted that all components of alliance are similarly related to the ability to build deep and trust-based relations with others (correlation between the dimensions of alliance and positive relations with others). The working alliance accompanies agency—which is crucial for recovery—and coping with complex environmental factors (correlation between the dimensions of alliance and environmental mastery); it also accompanies the feeling that the search for a further path of development and the challenges undertaken will lead to an increase in personal abilities (correlation between the dimensions of alliance and personal growth). This is consistent with previous findings. Ryff (48) stresses that what is crucial for the improvement of health and for human development is an increase in the sense of self-directedness and the ability to transform the environment in accordance with one's values and needs. It turns out that that a properly set-up alliance makes it possible to organize the therapeutic space in such a way as to support the process of intense work on the significant conditions of recovery and to facilitate these conditions.

Two other dimensions, autonomy and self-acceptance, are also related to the quality of the therapeutic alliance, though less strongly so. Thus, the working alliance—understood, after all, as an optimally built relationship in the process of psychotherapy—turns out to co-occur with the main characteristics of mental health: with a positive but also realistic attitude toward oneself, and with autonomy, enabling the effective intrinsic regulation of behavior. The therapeutic relationship seems to be an important stabilizing condition of maturation and development despite the fact that what is often the case, particularly in the psychotherapeutic process, is a fluctuating increase in the subjective sense of discomfort or the subjective experience of various difficulties.

The alliance sometimes accounts for a considerable percentage of the variance in scores on the explained variables measured in the study. The analyses yielded higher estimates in this respect compared to earlier findings (20, 27).

To sum up, the seven hypotheses tested based on the results of statistical analyses were supported and accepted. The actual relationship between the quality of working alliance and psychological wellbeing proved to be the strongest. The relations of the therapeutic alliance to satisfaction with life and the quality of flourishing in life are weaker, though their values are moderate.

The therapeutic alliance is an important factor accompanying the positive outcomes of psychotherapy, operationalized by means of subjective indicators—namely, wellbeing.

The relations of the therapeutic alliance to wellbeing, life satisfaction, and flourishing vary slightly depending on who is the source of information about the quality of the alliance: the patient alone or the patient and the psychotherapist (weighted evaluation by two individuals). If the alliance is evaluated by the patient alone, the relations are stronger.

### Constraints on Generality

Various limitations of the present study should be mentioned. In future studies the sample size should be increased, so that empirical support for SEM models can be stronger. Researchers should make sure that people with different characteristics in terms of extraneous variables are strongly represented, so that analyses taking their the impact of these variables into account can be performed. The current sample was too small and too heterogeneous to allow for distinguishing homogeneous subgroups of subjects. It becomes necessary in the future to identify the potential moderators of the analyzed relationships. What would also be valuable is longitudinal analyses, which, using at least two measurements performed at different stages of working alliance consolidation, could determine the dynamics of the relationships of alliance to wellbeing, satisfaction, and flourishing. This study had a cross-sectional design, and the treatment relationship and alliance may have fluctuated over time in ways that this kind of design does not detect. Given these limitations, the research project should be continued.

## Data Availability Statement

The raw data supporting the conclusions of this article will be made available by the authors, without undue reservation.

## Ethics Statement

The studies involving human participants were reviewed and approved by the Research Ethics Board at the Maria Grzegorzewska University (APS) in Warsaw. Written informed consent to participate in this study was provided by the participants' legal guardian/next of kin.

## Author Contributions

The author confirms being the sole contributor of this work and has approved it for publication.

## Funding

This research results presented in this article come from research project BSTP 32/19-I, financed by the Maria Grzegorzewska University.

## Conflict of Interest

The author declares that the research was conducted in the absence of any commercial or financial relationships that could be construed as a potential conflict of interest.

## Publisher's Note

All claims expressed in this article are solely those of the authors and do not necessarily represent those of their affiliated organizations, or those of the publisher, the editors and the reviewers. Any product that may be evaluated in this article, or claim that may be made by its manufacturer, is not guaranteed or endorsed by the publisher.
